# An Unusual Case of Neuroendocrine Carcinoma of the Gastro-Esophageal Junction Complicated by Syndrome of Inappropriate Antidiuretic Hormone Secretion

**DOI:** 10.7759/cureus.79807

**Published:** 2025-02-28

**Authors:** Asmita Kulshrestha, Deeksha Jawale, Ankit Sharma, Priyanka Bhoj, Amitabh Pandagle

**Affiliations:** 1 Department of Radiation Oncology, Cama and Albless Hospital, Mumbai, IND; 2 Department of Surgical Oncology, Tata Memorial Hospital, Mumbai, IND

**Keywords:** esophageal cancer (ec), hepatic metastasis, neuroendocrine carcinoma(nec), paraneoplastic syndromes, supportive and palliative care

## Abstract

Cancers of the esophagus tend to be aggressive with a poor prognosis. Neuroendocrine carcinoma (NEC) of the esophagus is very rare and currently does not have any established treatment regimens. This is in part due to its rare prevalence. This case report presents an unusual case of distal esophageal NEC, which was complicated by liver metastasis and paraneoplastic syndrome.

A 56-year-old postmenopausal woman came with complaints of weight loss, pain in the epigastric region, and intermittent dysphagia to solids for three months. An endoscopy revealed a 2 cm x 1 cm ulceronodular lesion located at the gastroesophageal junction. The biopsy showed NEC on histological examination, and the immunohistochemistry was positive for multiple neuroendocrine markers. A positron emission tomography (PET) scan revealed a stage IV metastatic disease that had spread to the liver as well as gastrohepatic and peri-gastric lymph nodes.

Subsequently, the patient received palliative intent chemotherapy with Etoposide and Cisplatin for four cycles, which resulted in a partial response. Due to this, she was advised four more cycles of chemotherapy, but after two cycles, she developed signs of the syndrome of inappropriate antidiuretic hormone secretion (SIADH). Following management with hypertonic saline, her health deteriorated, resulting in death within one year of diagnosis.

NECs of the esophagus tend to be detected at later stages and show a worse prognosis and disease course than squamous cell carcinoma or adenocarcinoma of the esophagus. With improvements in investigation methods, an increase in the detection of NEC in the esophagus can be expected in the future. Early diagnosis can help overall survival and boost quality of life. Further research and clinical trials are needed to assess ideal treatment plans.

## Introduction

Esophageal cancer is often associated with a poor prognosis. Globally, it is the 11th most common cancer diagnosed and the seventh leading cause of cancer-related death. The incidence is slightly higher in developed countries, with men being affected two to three times more than women. As a result, mortality rates are also greater among men [1].

The mean age of diagnosis ranges between 60 to 70 years, and the vast majority of esophageal malignancies are either squamous cell carcinoma (SCC) or adenocarcinoma (AC). The origin of primary neuroendocrine neoplasm of the esophagus (NNE) varies, with East Asian populations having NNEs in the middle of the esophagus and Western demographics exhibiting distal esophageal predominance [2]. NNEs are a rare and aggressive entity with a reported incidence between 0.04% and 1% [3]. Due to this uncommon histological diagnosis, prospective clinical trials to determine optimal management strategies with a multidisciplinary approach are still ongoing.

We present an unusual case of distal esophageal neuroendocrine carcinoma with liver metastasis at the outset, which was additionally worsened by the development of a paraneoplastic syndrome. Due to the aggressive nature and advanced stage of the disease, the patient succumbed to it within a year of diagnosis.

This article was previously presented as a poster at Oncovision 2025 on January 19, 2025.

## Case presentation

A 56-year-old postmenopausal woman came to our department with complaints of intermittent pain in the epigastric region and dysphagia to solids for three months. She had well-controlled hypertension but had lost more than 10% of her body weight (6 kg) in this period due to difficulty in oral intake. An esophagogastroduodenoscopy was done, which revealed a 2 cm x 1 cm ulceronodular lesion at the gastroesophageal junction (GEJ), from which a biopsy was taken.

**Figure 1:**
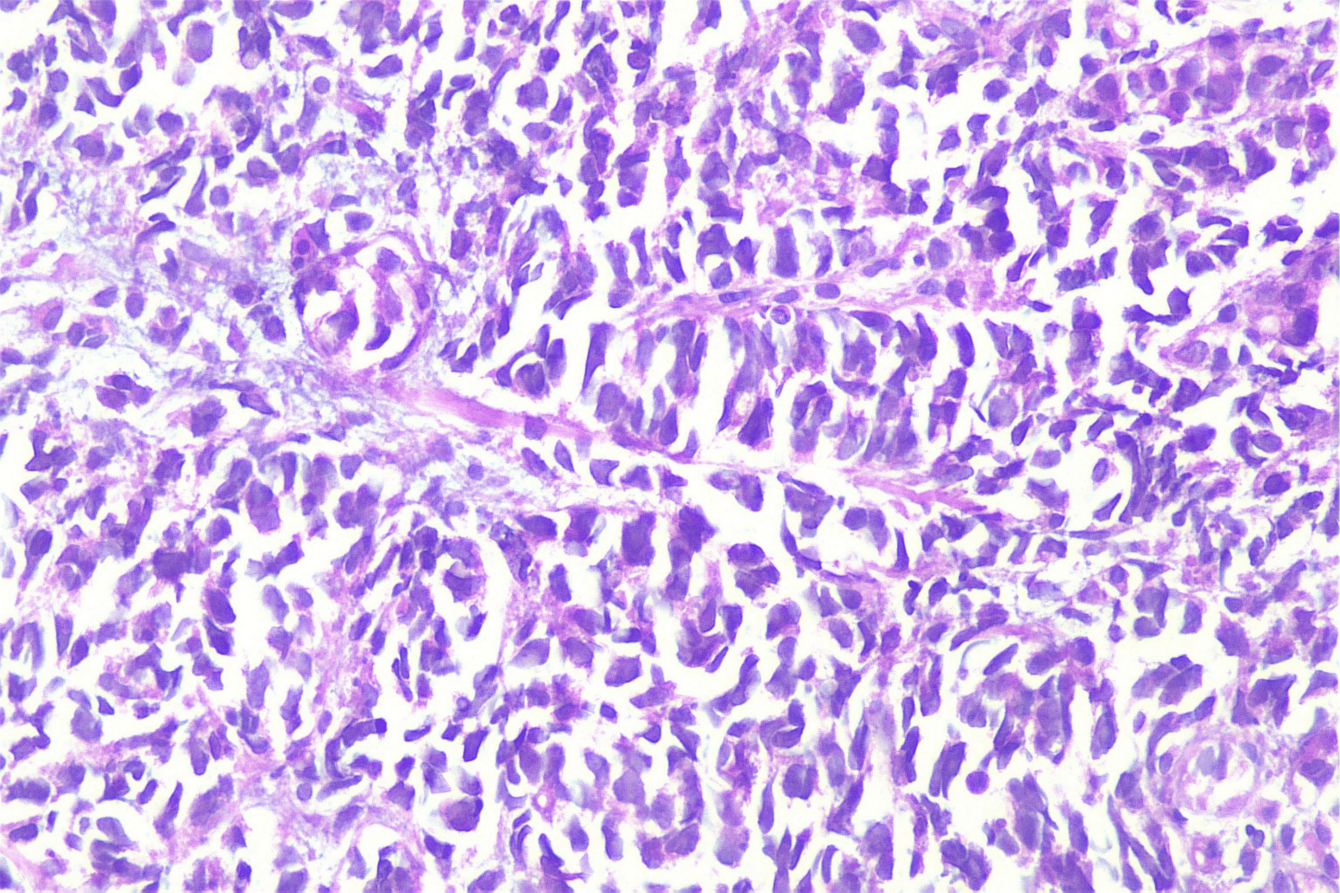
Hematoxylin and eosin staining of the tumor sample at 400x magnification showing neuroendocrine carcinoma Deeper mucosa showing atypical hyperchromatic cells with attempted gland formation.

**Figure 2:**
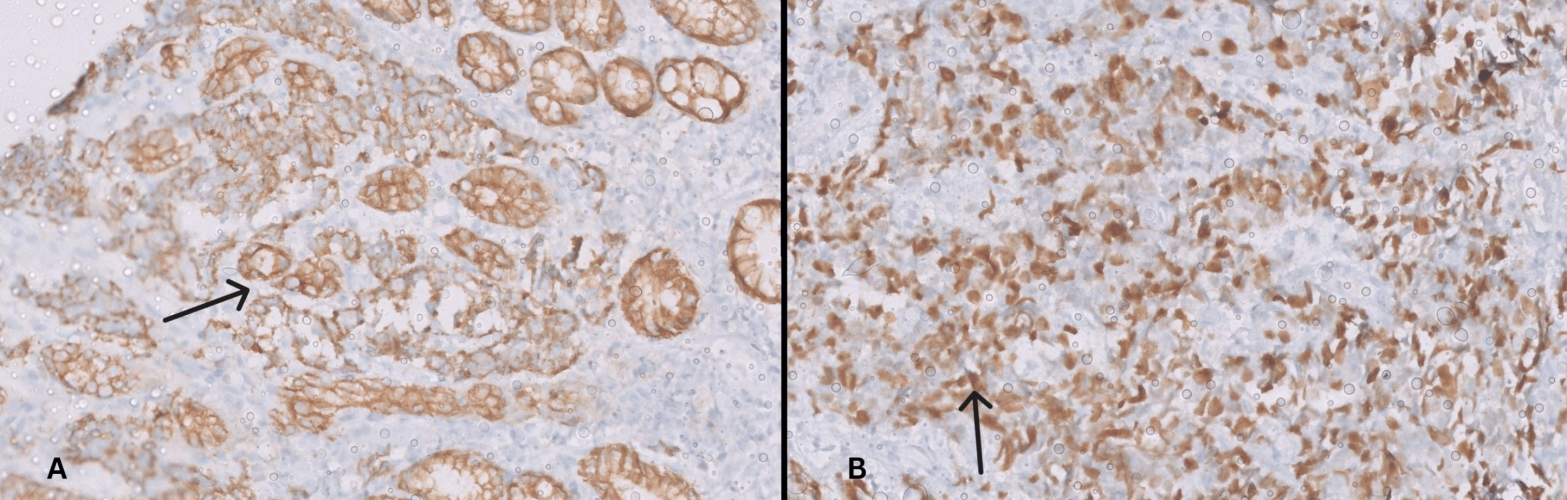
Tumor showing positive AE1/AE3 staining (A) and Mib-1 immunohistochemistry positivity (B), both at 100x magnification

On histopathological examination, the lesion was diagnosed as neuroendocrine carcinoma (Figure 1), which was positive for insulinoma-associated protein one (INSM1), cytokeratin AE1 and AE3 (Figure 2A). The atypical cells in the deeper mucosa of the sample showed hyperchromatic cells and were focally positive for chromogranin as well as weakly positive for synaptophysin. The Mib-1 index (cell proliferation marker) was 70% (Figure 2B).

A positron emission tomography (PET) scan showed asymmetrical circumferential wall thickening involving the GEJ and fundus of the stomach, measuring 4.5 cm x 3.9 cm x 3.8 cm. Enlarged gastrohepatic and peri-gastric lymph nodes were noted, the largest node measuring 2.7 cm x 2 cm. There was resultant dilatation of the distal esophagus, and hepatic lesions were present in segments IV, VI, VII, and VIII, with the largest lesion measuring 2.3 cm x 1.8 cm. The staging was T4aN2M1. Tumor markers and blood test results at the time of diagnosis are given in Table 1.

**Table 1:** Tumor marker values and blood investigations of the patient at the time of diagnosis CEA: Carcinoembryonic antigen; CA-19.9: Cancer antigen 19.9; SGOT: Serum glutamic oxaloacetic transaminase; SGPT: Serum glutamate pyruvate transaminase

Investigation	Patient’s values	Reference values
Serum CEA	165 ng/mL	0-5 ng/mL
Serum CA-19.9	174 U/mL	0-37 U/mL
Total Bilirubin	1.0 mg/dL	0.3-1.2 mg/dL
SGOT	28 U/L	15-40 U/L
SGPT	30 U/L	10-49 U/L

The patient’s treatment was planned with palliative intent due to the metastatic presentation of the disease. She was started on four cycles of palliative chemotherapy, consisting of Etoposide 120 mg (100 mg/m^2^) and Cisplatin 40 mg (33 mg/m^2^) on days one, two, and three every 21 days. A post-chemotherapy PET scan was done that depicted partial response in the primary tumor as well as gastro-hepatic and peri-portal lymph nodes, complete resolution of liver lesions, but new onset of metastatic right upper and lower paratracheal lymph nodes suggestive of progressive disease.

As a result, she was planned for four additional cycles of chemotherapy with Etoposide 120 mg (100 mg/m^2^) on days one, two, and three and Carboplatin 500 mg (area under curve five) on day one, every 21 days. However, the patient developed fatigue, headache, bilateral pedal edema, and confusion over three days after the second cycle. Blood and urine investigations showed the following results (Table 2).

**Table 2:** Blood and urine investigation results

Investigation	Patient’s values	Reference values
Serum sodium	119 mmol	135-145 mmol
Serum potassium	4.2 mmol	3.5-5 mmol
Serum creatinine	0.6 mg/dL	0.5-1.1 mg/dL
Serum osmolality	258 mOsm/kg	>275 mOsm/kg
Urine osmolality	125 mOsm/kg	<100 mOsm/kg
Urine sodium	41 mmol/L	<20 mmol/L

The fluid intake of the patient was around 2 liters per day, with a reduced urine output of less than 500 ml in 24 hours. A provisional diagnosis of the syndrome of inappropriate antidiuretic hormone secretion (SIADH) was made. On treatment with hypertonic saline (3% sodium chloride), her symptoms improved, and her laboratory results returned to normal. Additional computed tomography (CT) scans done to assess further treatment options revealed the progression of the disease, leading to an increase in the severity of her cancer symptoms. The patient subsequently experienced critical deterioration in her health, leading to a fatal outcome.

## Discussion

As per the World Health Organization's 2019 classification of tumors of the digestive system, neuroendocrine neoplasms of the gastrointestinal tract have been divided into neuroendocrine tumors (NET) and neuroendocrine carcinomas (NEC). NETs are further subdivided into grades G1, G2, and G3. Low-grade NETs, grade 1 or 2, are defined as well-differentiated tumors with a Ki-67 index of less than 20%, while high-grade tumors with a Ki-67 index of more than 20% are classified as grade 3. Grade 3 tumors can be well-differentiated or poorly differentiated. Poorly differentiated Grade 3 tumors are referred to as NECs. They grow faster, are more aggressive, and require different treatments than lower-grade NETs [4,5].

Although specific risk factors for NEC esophagus have limited research, family history, a high body mass index, and diabetes have been cited as the predominant risk factors for neuroendocrine malignancies in other organs, with cigarette smoking and alcohol consumption showing an association at some sites [6]. Positive neuroendocrine markers like chromogranin A, synaptophysin, and CD56 (neural cell adhesion molecule) are needed for the diagnosis. Additionally, a Ki-67 index above 20% is required, and some test positive for neural markers like neuron-specific enolase or S100 [7].

Symptoms include dysphagia, pain, retrosternal discomfort, weight or appetite loss, vomiting, melena, hematemesis, gastroesophageal reflux disease, or symptoms associated with metastasis [8]. NECs have a larger tumor size and poorer prognosis in comparison to ACs and SCCs of the esophagus. Their aggressive course, especially in cases of high-grade tumors, results in a large proportion of patients presenting with advanced stages at the time of diagnosis, as was the case for our patient [9].

Paraneoplastic syndromes (PNS) in NNE can be seen due to the production and secretion of physiologically active hormones and peptides by the tumor. Endocrine PNS associated with NNE are SIADH and humoral hypercalcemia of malignancy. SIADH is the most common cause of hyponatremia in malignancy. This condition is defined by the unregulated secretion of antidiuretic hormone (ADH) from the pituitary gland, non-pituitary sources, or its sustained effects on vasopressin receptors [10,11].

The diagnosis of SIADH involves six essential criteria: serum osmolality of less than 275 mOsm/kg; urine osmolality of more than 100 mOsm/kg; clinical euvolemia, indicated by the lack of any signs and symptoms of volume loss; raised urine sodium concentration higher than 30 mmol/L (in the presence of normal salt and water intake); absence of other potential causes of euvolemic hypo-osmolality such as severe hypothyroidism, adrenal insufficiency and abnormal renal function and lastly, no recent diuretic intake [11].

SIADH can be caused by malignancies (with 70% of SIADH due to cancer being attributable to small-cell lung cancer), pulmonary and nervous system infections, disorders of the nervous system, drugs like anti-depressants (mainly selective serotonin reuptake inhibitors), antipsychotics, anticonvulsants, and vinca alkaloids. It can also be hereditary due to mutation of vasopressin receptors [12].

Differentials showing hyponatremia include cerebral salt wasting due to intracranial pathologies like sub-arachnoid hemorrhage, antidiuretic-hormone analog or thiazide diuretic use, severe hypothyroidism (dilutional hyponatremia), adrenal insufficiency (accompanied by hyperkalemia), hyperglycemia (translational hyponatremia), advanced kidney failure and low salt intake [13].

SIADH is associated with symptoms like confusion, fatigue, headache, nausea, and even coma or death in severe cases [10]. Treatment guidelines for acute, symptomatic hyponatremia is the administration of a bolus of 3% sodium chloride, accompanied by rigorous monitoring of serum sodium levels to prevent overcorrection. Vasopressin-2 receptor antagonists, or vaptans, can also be utilized as they don't require fluid restriction and successfully raise sodium levels [14].

As for the treatment of NECs, only surgery or radiotherapy alone had an abysmal five-year survival rate of 0%, the median survival time being less than a year. In contrast, surgery, when combined with chemotherapy or radiotherapy, achieved a five-year survival rate of 27.2% and a median survival time of 22 months [15]. Surgery with chemoradiotherapy is currently the mainstay of treatment for localized disease, leading to longer survival and better quality of life. The therapeutic effect of chemotherapy is limited; however, in advanced stages where surgical resection is not possible, it becomes the first choice [16].

## Conclusions

With improved investigation modalities, a rise in the detection of rare malignancies like neuroendocrine carcinoma of the esophagus can be expected. Due to its aggressive disease course and advanced presentation at diagnosis, early identification and management are crucial to achieving a better prognosis.

Our case had complications, with the patient showing only a partial response to palliative treatments. Currently, a combination of surgery, chemotherapy, and radiotherapy is used for treatment, but clinical trials to assess optimal treatment plans are needed and can help increase overall survival.
